# Enhanced skin cancer diagnosis through grid search algorithm-optimized deep learning models for skin lesion analysis

**DOI:** 10.3389/fmed.2024.1436470

**Published:** 2024-11-07

**Authors:** Rudresh Pillai, Neha Sharma, Sheifali Gupta, Deepali Gupta, Sapna Juneja, Saurav Malik, Hong Qin, Mohammed S. Alqahtani, Amel Ksibi

**Affiliations:** ^1^Chitkara University Institute of Engineering and Technology, Chitkara University, Rajpura, Punjab; ^2^CSE(AI), KIET Group of Institutions, Ghaziabad, India; ^3^Department of Environmental Health, School of Public Health, Harvard University, Boston, MA, United States; ^4^Department of Pharmacology and Toxicology, University of Arizona, Tucson, AZ, United States; ^5^School of Data Science, Department of Computer Science, Old Dominion University, Norfolk, VA, United States; ^6^University of Tennessee at Chattanooga, Chattanooga, TN, United States; ^7^Department of Radiological Sciences, College of Applied Medical Sciences, King Khalid University, Abha, Saudi Arabia; ^8^BioImaging Unit, Space Research Centre, University of Leicester, Leicester, United Kingdom; ^9^Department of Information Systems, College of Computer and Information Sciences, Princess Nourah Bint Abdulrahman University, Riyadh, Saudi Arabia

**Keywords:** deep learning (DL), Convolutional Neural Network (CNN), grid search algorithm, binary classification, multiclass classification, skin cancer, skin lesions

## Abstract

Skin cancer is a widespread and perilous disease that necessitates prompt and precise detection for successful treatment. This research introduces a thorough method for identifying skin lesions by utilizing sophisticated deep learning (DL) techniques. The study utilizes three convolutional neural networks (CNNs)—CNN1, CNN2, and CNN3—each assigned to a distinct categorization job. Task 1 involves binary classification to determine whether skin lesions are present or absent. Task 2 involves distinguishing between benign and malignant lesions. Task 3 involves multiclass classification of skin lesion images to identify the precise type of skin lesion from a set of seven categories. The most optimal hyperparameters for the proposed CNN models were determined using the Grid Search Optimization technique. This approach determines optimal values for architectural and fine-tuning hyperparameters, which is essential for learning. Rigorous evaluations of loss, accuracy, and confusion matrix thoroughly assessed the performance of the CNN models. Three datasets from the International Skin Imaging Collaboration (ISIC) Archive were utilized for the classification tasks. The primary objective of this study is to create a robust CNN system that can accurately diagnose skin lesions. Three separate CNN models were developed using the labeled ISIC Archive datasets. These models were designed to accurately detect skin lesions, assess the malignancy of the lesions, and classify the different types of lesions. The results indicate that the proposed CNN models possess robust capabilities in identifying and categorizing skin lesions, aiding healthcare professionals in making prompt and precise diagnostic judgments. This strategy presents an optimistic avenue for enhancing the diagnosis of skin cancer, which could potentially decrease avoidable fatalities and extend the lifespan of people diagnosed with skin cancer. This research enhances the discipline of biomedical image processing for skin lesion identification by utilizing the capabilities of DL algorithms.

## 1 Introduction

The body's largest organ, the skin (1), is the soft, flexible outer tissue separating a human body's internal systems and organs from its environment. It has a complex structure which is further divided into three layers: the epidermis, the dermis, and the hypodermis. It serves three major tasks: Protection, Sensation, and Regulation. It protects the body from heat, light, injury, and infection. It also assists in regulating the temperature of the human body (2) and serves as a sensory organ, providing a sense of touch to humans. As it covers the entire human body, it has a total surface area of 20 square feet, making it an essential human organ. Various internal and external factors, such as aging, sun exposure, infections, and injuries, lead to skin lesions (3). They are characterized as any anomaly in the skin's color, texture, or appearance, including lesions, lumps, or bumps. Based on the underlying causes, skin lesions can be categorized as infectious, neoplastic, or inflammatory. Skin lesions can be categorized based on their appearance and where they occur. A skin lesion can be categorized as benign or malignant (4) based on whether the lesion develops into cancer and spreads to other body parts. A lesion is considered benign when the cells do not invade other tissues and remain contained within the lesion. Malignant lesions contain cancerous cells that spread to other tissues and cause significant harm to the infected regions. Thus, it is essential to categorize skin lesions timely and accurate to detect whether a lesion is a form of skin cancer.

Skin cancer (5), the most common category of cancer (6), refers to abnormal cell duplication caused by DNA mutation. This condition results when the DNA of skin cells gets damaged due to UV rays (7) from the sun or artificial sources for prolonged periods. This leads to the damaged skin cells growing abnormally to form tumors. Skin cancer can be categorized into Basal Cell Carcinoma (BCC), Squamous Cell Carcinoma (SCC), and Melanoma (8). BCC and SCC are the two most frequent skin cancer types. BCC affects the basal cells of the lower part of the epidermis, causing lesions to be formed on the skin's surface. SCC is due to the abnormally increased development of squamous cells in the epidermis due to prolonged exposure to sunlight. The least common type of skin cancer, which is melanoma, is the most risky and invasive form of skin cancer with the highest probability of fatality. Also known as 'black tumor,' it accounts only for 1% of all cancers but is the cause of most significant of the demises caused by skin cancer. The WHO, in its 'World Cancer Report: Cancer Research for Cancer Development, (9) stated that every year, over 13 lakh cases of melanoma and around 25 lakh cases of non-melanoma are reported worldwide annually e, accounting for every third cancer diagnosis. Traditionally, examining the skin visually and doing a biopsy are conventional ways of finding skin lesions. The appearance of the skin lesion is commonly examined by a dermatologist, who may also study the lesion's anatomy using a dermatoscope, a portable magnifying instrument (10). A tissue sample is detached in biopsy and then sent to a laboratory for investigation to help identify the skin lesion's presence. Although these approaches are viable, they are laborious and arbitrary, resulting in many false positives and negatives. In medical image analysis (11), machine learning (ML) procedures (12), specifically DL architectures (13), have made significant advancements recently. DL is a kind of ML that uses massive datasets to train neural networks (NN) to recognize patterns and predict future outcomes. DLNNs, called Convolutional Neural Networks (CNNs), are exceptionally proficient at image identification and classification tasks. This research aims to develop a system of fully automated CNNs for multi-classifying skin lesions using datasets developed by ISIC (14). For this research, the classification of the images was divided into three Tasks. Three different CNNs were implemented for the three different classification Tasks. For Task 1, binary classification of images was carried out to ascertain whether Skin Lesions were detected. For Task 2, binary classification of images was carried out to ascertain whether the lesion detected was benign or malignant. For Task 3, multi-classification of images was carried out to confirm one of the seven types of skin lesions: Actinic Keratosis & Intraepithelial Carcinoma/Bowen's Disease (AKIEC), Basal Cell Carcinoma (BCC), Benign Keratosis-like Lesions (BKL), Dermatofibroma (DF), Melanoma (MEL), Melanocytic Nevi (NV), & Vascular Lesions (VASC). A separate dataset was created for each task taken from the ISIC Archive. The dataset is divided into two sets: train and test. After training, the performance of the proposed CNN models was evaluated. Performance evaluation was achieved using methods such as Loss Analysis, Accuracy Analysis, and Confusion Matrix. The Confusion Matrix (15) is a square table representation of the true labels and predicted labels of the images by a CNN model. It is used to derive various performance characteristics, including Accuracy, Precision, Recall/Sensitivity, F1 Score, and Specificity.

The significant contributions of this research are presented as follows:

- A CNN model-based approach is used to diagnose skin lesions. Three CNN models are presented for three classification tasks: detecting a skin lesion, determining if the lesion is benign or malignant, and categorizing the skin lesion by kind.
- To train and evaluate the proposed CNN models, images from the ISIC Archive were used to create three datasets with class-annotated images based on the three separate classification tasks. Data Augmentation was used to increase the variety of the datasets. The datasets were divided into two sets for training and testing the models.
- The CNN models' performance was assessed using Analysis Plots for Loss and Accuracy during training and testing and the Confusion Matrix. The Confusion Matrix is utilized to calculate performance metrics such as Accuracy, Precision, Recall/Sensitivity, and F1 Score, which provide a complete picture of the proposed CNN model for the intended classification job.

The remaining sections of the research paper are as follows: Section II explores previous research studies conducted in this domain. The methodology employed to carry out the proposed research is described in Section III. The results of the proposed study have been emphasized in Section IV. The concluding thoughts on the proposed research effort and its potential scope are provided in Section V.

## 2 Literature work

Dorj et al. (16) implemented an SVM to classify skin diseases. The authors utilized an AlexNet transfer learning (TL) model to extract features. The dataset employed for the study consisted of 3,753 images procured from the internet. The research achieved a classification accuracy of 92.3% for AKIEC, 91.8% for BCC, 95.1% for SCC, and 94% for MEL. Maron et al. (17) proposed using a customized CNN model with 112 dermatologists to classify skin diseases. The images were obtained from the HAM10000 dataset, supplemented with more images from the ISIC archive. The input dataset consisted of 11,444 dermatoscopic images of various skin-related diseases, including multiple types of skin lesions. Amin et al. (18) performed skin lesion segmentation by utilizing the Otsu algorithm. Pre-processing of images was performed to resize the images. The authors merged different datasets to generate a novel dataset of 7,849 images. A fusion of AlexNet and VGG16 features was implemented to classify images of MEL and BCC. The research attained an accuracy of 99%, sensitivity of 99.52%, and specificity of 98.41%. Hekler et al. (19) utilized images of MEL and NV to train and evaluate the ResNet50 TL model for examining label noise effects. The input dataset consisted of 804 images of MEL and NV procured from a combination of HAM10000 and ISIC Archive. Accuracy was evaluated for two types: For medical applications, the accuracy attained was 75%, and for biopsy, the accuracy achieved was 74%. The authors observed that the DL approach was extremely superficial and recommended biopsy-verified images to reduce the effect of label noise. Mahbod et al. (20) proposed a three-stage fusion technique combined with image downsampling and skin lesion cropping. The input dataset consisted of 12,927 dermatoscopic images of skin lesions. A CNN model was implemented to classify skin diseases. The research achieved an accuracy of 86.2%. However, the proposed research presented some limitations as significant training time was required for the many implemented sub-models. Han et al. (21) suggested a model for skin lesion classification. The dataset was formed by procuring dermoscopic skin lesion images from various hospitals, with 2,844 images. The RCNN architecture was implemented for classification into two categories based on the type of carcinoma detected, i.e., BCC and SCC. The research achieved an AUC score of 0.91. Masni et al. (22) proposed an analysis of TL models to classify three types of skin lesions. The dataset was taken using the ISIC 2017 dataset and consisted of 2,750 dermatoscopic images of skin lesions. A comparison between InceptionV3, ResNet50, Inception-ResNetV2, and DenseNet201 TL models was presented based on the classification of the dataset into NV, MEL, and AKIEC. The TL models' accuracies were: InceptionV3-−81.29%, ResNet50-−81.57%, Inception-ResNetV2-−81.34%, and DenseNet201–73.44%. Polat et al. (23) presented a CNN design to classify skin lesions into seven classes. The dataset, which consisted of a total of 10,015 images, was used for input. The CNN model attained 77% accuracy. Duggani et al. (24) employed a deep learning approach by proposing and implementing a customized CNN design to classify skin disease. The dataset consisted of 200 images from the PH2 dataset. The CNN design was utilized to categorize the dataset into two types: MEL and NV. The authors observed that the CNN model attained 97.49% accuracy. Khan et al. (25) employed a deep learning approach by proposing and implementing a customized CNN design. The dataset consisting of 10,015 dermatoscopic images of distinct types of skin diseases was employed for the research study. The CNN design was used to categorize the seven types: NV, DF, MEL, AKIEC, BKL, BCC, and VASC. The research achieved 87% accuracy, 86% sensitivity, 87.01% precision, and 86.28% F1 score. Shetty et al. (26) presented research on classifying images into seven distinct forms of skin lesions. The authors observed that a customized CNN model achieved an accuracy of 95.18%. Anand et al. (27) proposed an analysis of the VGG16 model and a modified VGG16 TL model with added multiple fine-tuning layers for skin lesion detection. The input dataset consisted of 3,297 images procured from the internet. Data augmentation techniques were implemented for diversifying the dataset. The models were implemented to classify the images between benign and malignant classes. Several hyperparameters were optimized and compared for better performance. The authors observed that the modified VGG16 TL model achieved 90% accuracy. Anand et al. (28) employed a TL approach by employing an Xception TL model for the detection of skin lesions. The HAM10000 dataset consisting of 10,015 images was utilized as the input dataset. Data Augmentation techniques were implemented on the input dataset for diversification. The Xception TL model classified the input dataset images into seven types of skin diseases and achieved 96.40% results. Aldhyani et al. (29) utilized the DL approach for skin disease detection by proposing and implementing a lightweight dynamic kernel CNN. The HAM10000 was utilized as the input. The proposed CNN model consisted of dynamic-sized kernels, significantly reducing the number of trainable parameters. The authors observed an accuracy of 97.85%, achieved by the proposed CNN model. Nigar et al. (30) designed and proposed an Explainable approach. The dataset employed in the research consisted of 25,331 images from the ISIC 2019. The suggested XAI system was executed to classify dermatoscopic images into eight distinct types of skin lesions.

## 3 Proposed methodology

**Figure 1:**
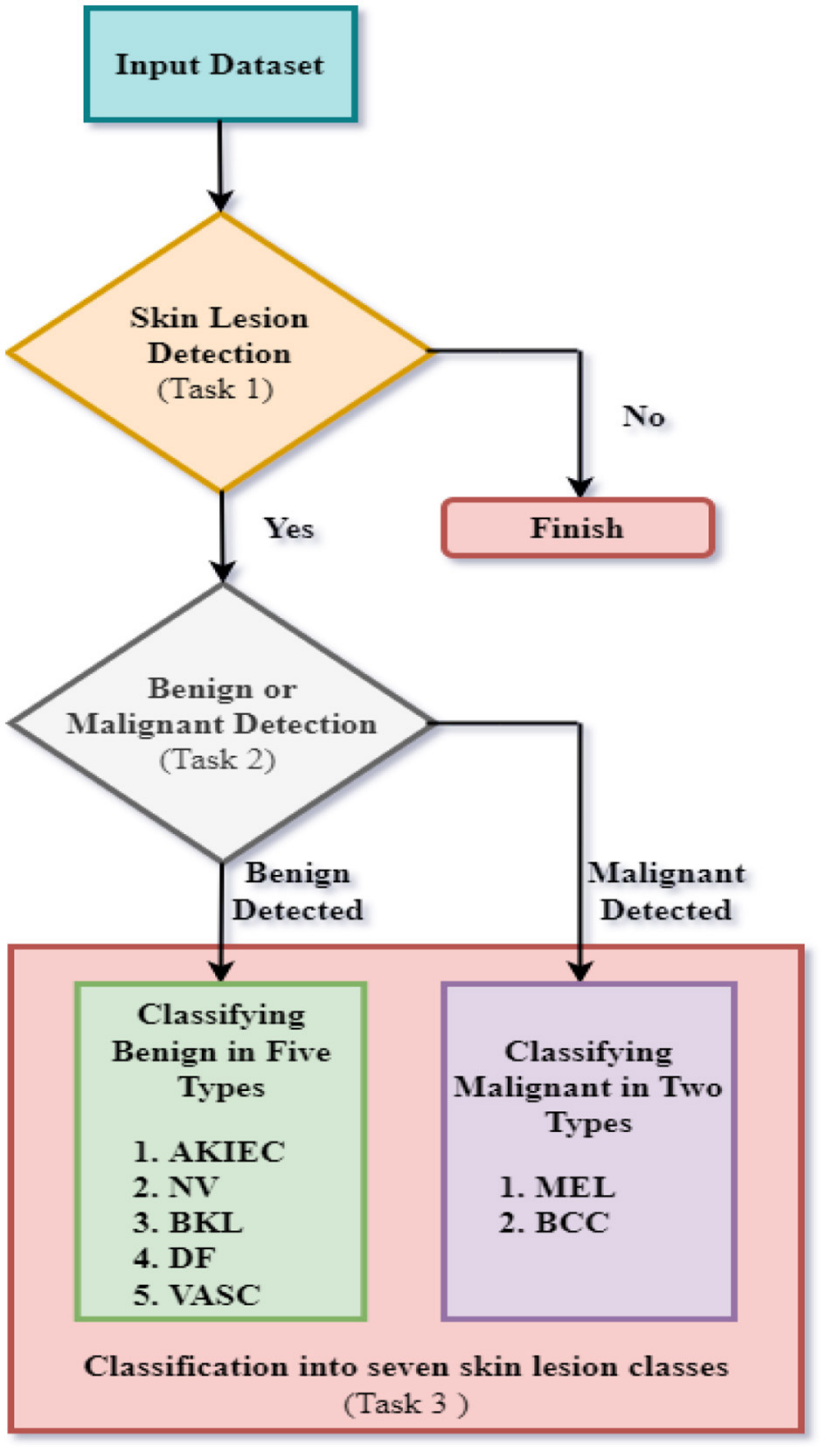
Flowchart for proposed methodology of complete diagnosis of skin lesions.

This research study proposes a fully automated system of CNN models for ultimately detecting a skin lesion to classify a particular type of skin lesion using datasets developed from the ISIC Archive. This is achieved by dividing the classification of images into three Tasks. Figure 1 represents the flow chart of the suggested research for the complete diagnosis of skin lesions.

The first task involves binary classification of images to ascertain whether skin lesions are in the images of the first dataset or not. The second task involves binary classification of images to classify images of the second dataset based on whether the skin diseases are benign or malignant. The third task involves multiclass classification of benign/malignant skin lesions according to further specific types, as shown in Figure 1. For task 3, seven skin lesion classes are taken as Actinic Keratoses and Intraepithelial Carcinoma/Bowen's Disease (AKIEC), Basal Cell Carcinoma (BCC), Benign Keratosis-like Lesions (BKL), Dermatofibroma (DF), Melanoma (MEL), Melanocytic Nevi (NV), and Vascular Lesions (VASC). The three tasks are accomplished using three distinct CNN models for each task. The proposed CNN designs were trained and tested using images from three distinct datasets formed from the ISIC Archive. First, the classification of images of the first dataset was implemented for the detection of skin lesions utilizing the first proposed CNN architecture. Next, the second CNN design was implemented to categorize images of the second dataset to ascertain whether skin lesions are benign or malignant. Finally, the third CNN model was implemented to classify images into seven specific categories of benign/malignant skin lesions.

The use of three unique CNNs for three separate skin lesion classification tasks has several benefits: It is possible to tune each CNN for a specific task, enabling the customization of architecture and hyperparameter configurations to achieve optimal performance for the given classification problem. The pursuit of this specialism has the potential to enhance accuracy and increase the reliability of forecasts in many tasks. The use of a dedicated CNN for each task enables the model to concentrate on acquiring knowledge pertaining to the distinct characteristics associated with that particular activity while minimizing the influence of other tasks' intricacies. For instance, the CNN may be specifically constructed to differentiate between benign and malignant tumors by only emphasizing characteristics that are suggestive of malignancy. Rather than constructing a singular, intricate model to address various tasks, the use of distinct CNNs enables the development of more straightforward architectures that are more manageable and trainable.

Furthermore, this phenomenon may result in expedited training durations and reduced computational expenditures. The use of separate models for each task facilitates the comprehension of the decision-making process employed by each CNN. This may be of significant use in comprehending the behavior of the model, particularly in medical contexts where the capacity to provide explanations is of utmost importance. The use of distinct CNNs for various tasks may enhance the model's ability to generalize its performance over a wide range of datasets. This is because each network can effectively adapt to the unique intricacies and variations present in the data that are pertinent to its respective job. The use of distinct CNNs enables a modular methodology for the identification of skin lesions. Each model can undergo separate enhancements, updates, or replacements without causing any impact on the other models. This characteristic allows for flexibility in the maintenance and development of the system. The use of distinct CNNs for distinct tasks enables the isolation of errors in a particular model, hence facilitating the identification and resolution of difficulties within the classification process. Furthermore, this approach enables more focused debugging and improved refining of particular models.

### 3.1 Dataset description

The International Skin Imaging Collaboration (ISIC), with the primary aim of minimizing melanoma mortality through the facilitation of the administration of digital skin imaging, is an international bond between academics and the industry. The ISIC Archive archives readily accessible skin lesion images under the Creative Commons License. Dermoscopic images of specific skin lesions have been the archive's initial emphasis since they are intrinsically regulated due to the use of a specialized capture instrument and lack many of the privacy concerns of medical imaging. The images available through the archive are annotated with ground-truth diagnoses and further clinical metadata. This research study utilized annotated images from the ISIC Archive to form three distinct datasets for each classification task. The datasets contained various images for each task according to the classification tasks. The classes, number of images, and train-test splits for each classification task are presented in the following sub-sections.

**Figure 2:**
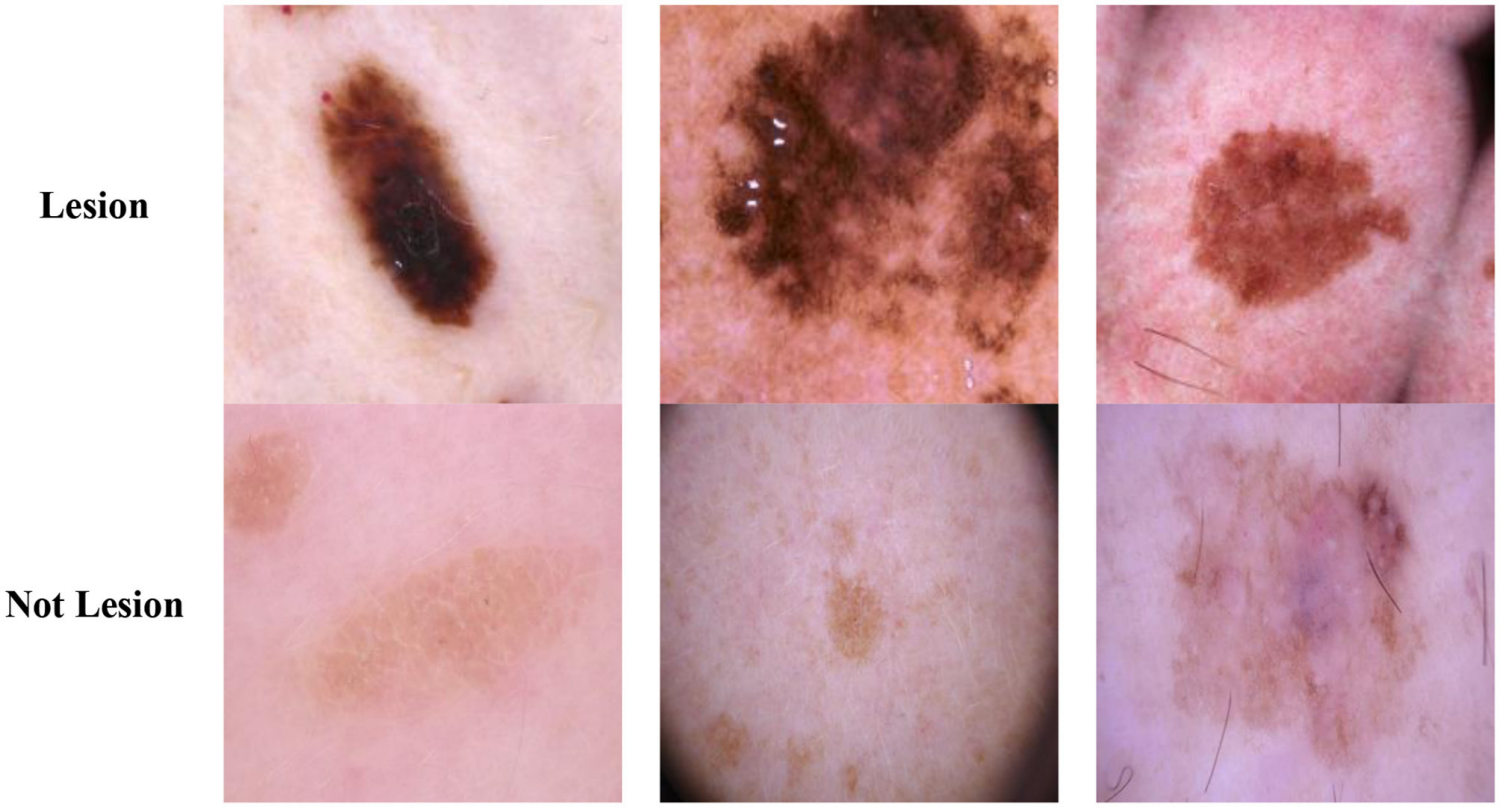
Classification task 1: skin lesion detection dataset images.

The Classification Task 1 Dataset consisted of images labeled with two classes according to Classification Task 1, which involved the detection of skin lesions. Thus, the dataset images were labeled according to whether skin lesions were detected. The dataset consisted of 17,806 images labeled Lesion and Not lesion. The input images' size was 224 by 224 pixels, and the number of channels was set at 3. The dataset was split into 12,464 images (70%) for the train set, 1,780 images (10%) for the validation set, and 3,561 images (20%) for the test set. Figure 2 displays the class labels for Classification Task 1 and some images from each class.

**Figure 3:**
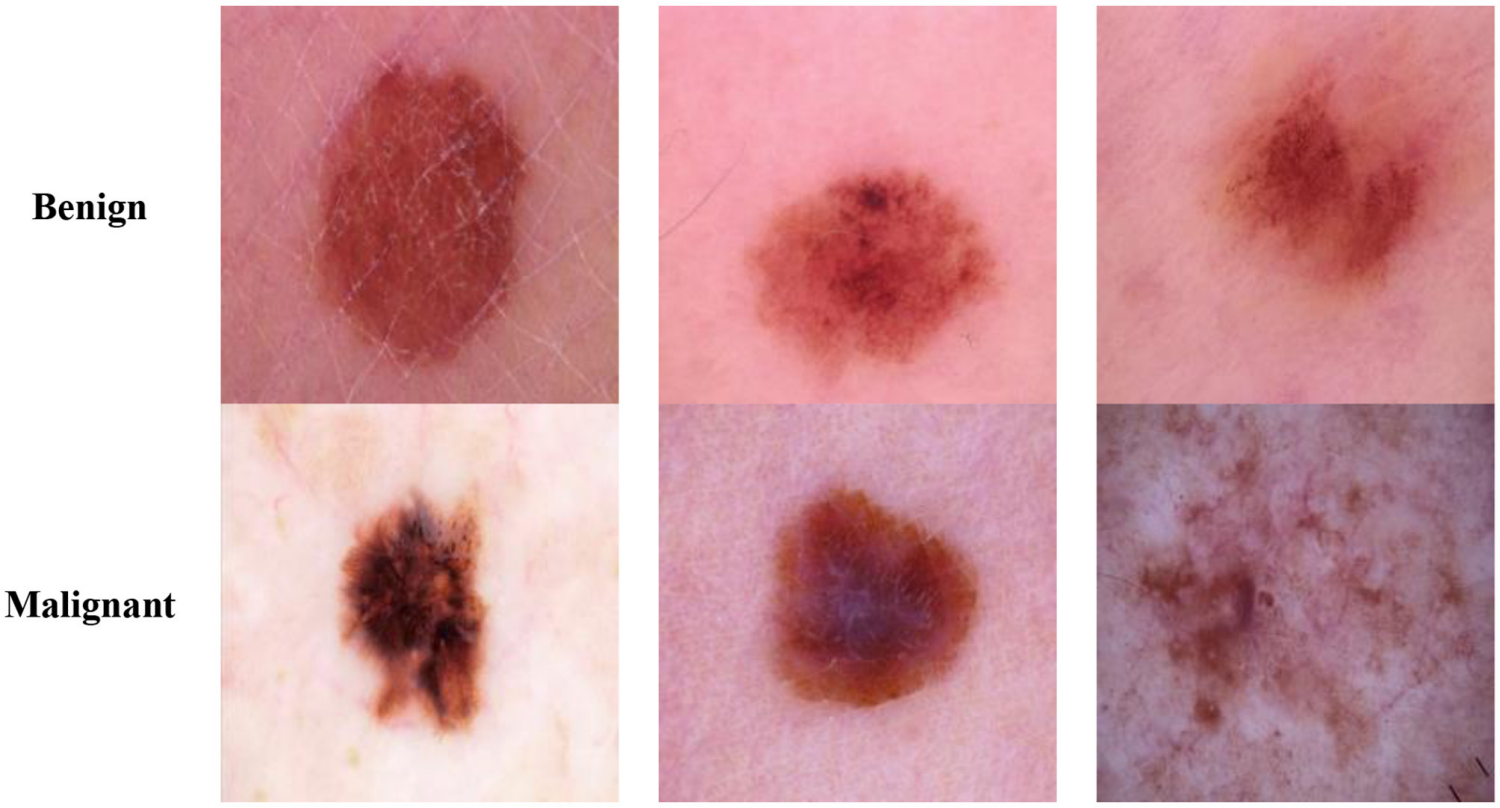
Classification task 2: classification of benign and malignant dataset images.

The Classification Task 2 Dataset consisted of images labeled with two classes according to Classification Task 2 for the classification of the skin lesions as Benign and Malignant. The dataset consisted of 3,297 images labeled with classes Benign or Malignant. The input images' size was 222 by 222 pixels, and the number of channels was set at 3. The database has been split into 2,307 images (70%) for the train set, 330 images (10%) for the validation set, and 660 images (20%) for the test set. Figure 3 displays the class labels for Classification Task 2 and some images from each class.

**Figure 4:**
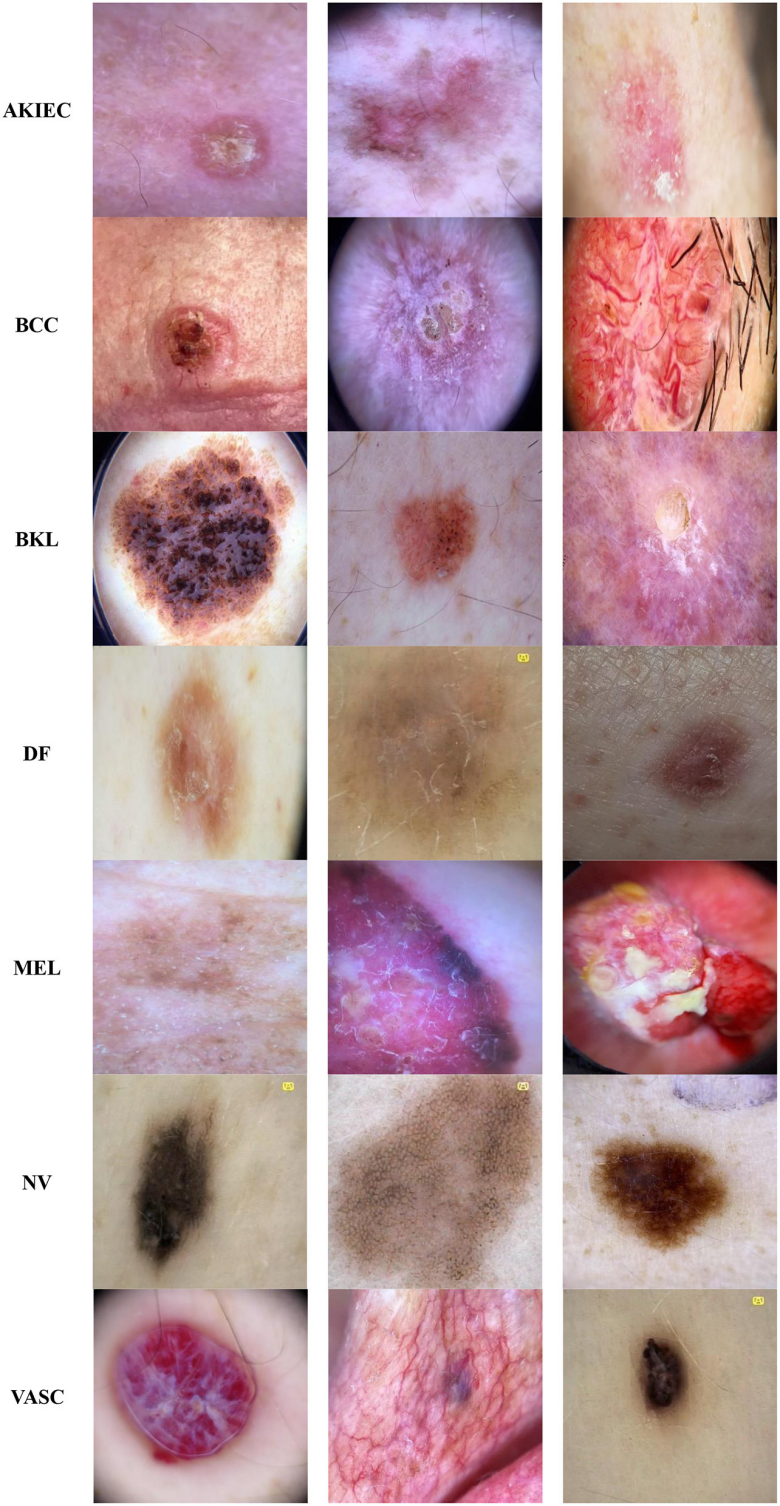
Classification task 3: benign/malignant skin lesion classification in seven classes dataset images.

The Classification Task 3 Dataset consisted of images labeled with two classes according to Classification Task 3 to categorize the skin diseases into seven categories: AKIEC, BCC, BKL, DF, MEL, NV, & VASC. The dataset consisted of 46,935 images. The input images were 28 by 28 pixels, and the number of channels was 3. The dataset was split into three sets consisting of 30,508 images (65%) for training, 4,693 images (10%) for validation, and 11,734 images (25%) for testing. Figure 4 displays the class labels for Classification Task 3 and some images of skin lesions.

Table 1 represents the various classification groups for each classification task involved in complete skin disease detection. The Classification Groups, Number of images for each group, and the total number of images for specific classification tasks are highlighted for each classification task.

**Table 1:** Number of skin lesion images for each dataset.

**Classification task**	**Classification group**	**Number of images for each group**	**Total number of images**
Classification 1	Lesion	8,903	17,806
	Not Lesion	8,903	
Classification 2	Benign	1,800	3,297
	Malignant	1,497	
Classification 3	AKIEC	6,696	46,935
	BKL	6,718	
	BCC	6,680	
	DF	6,658	
	MEL	6,692	
	NV	6,709	
	VASC	6,784	

### 3.2 Data augmentation

**Figure 5:**
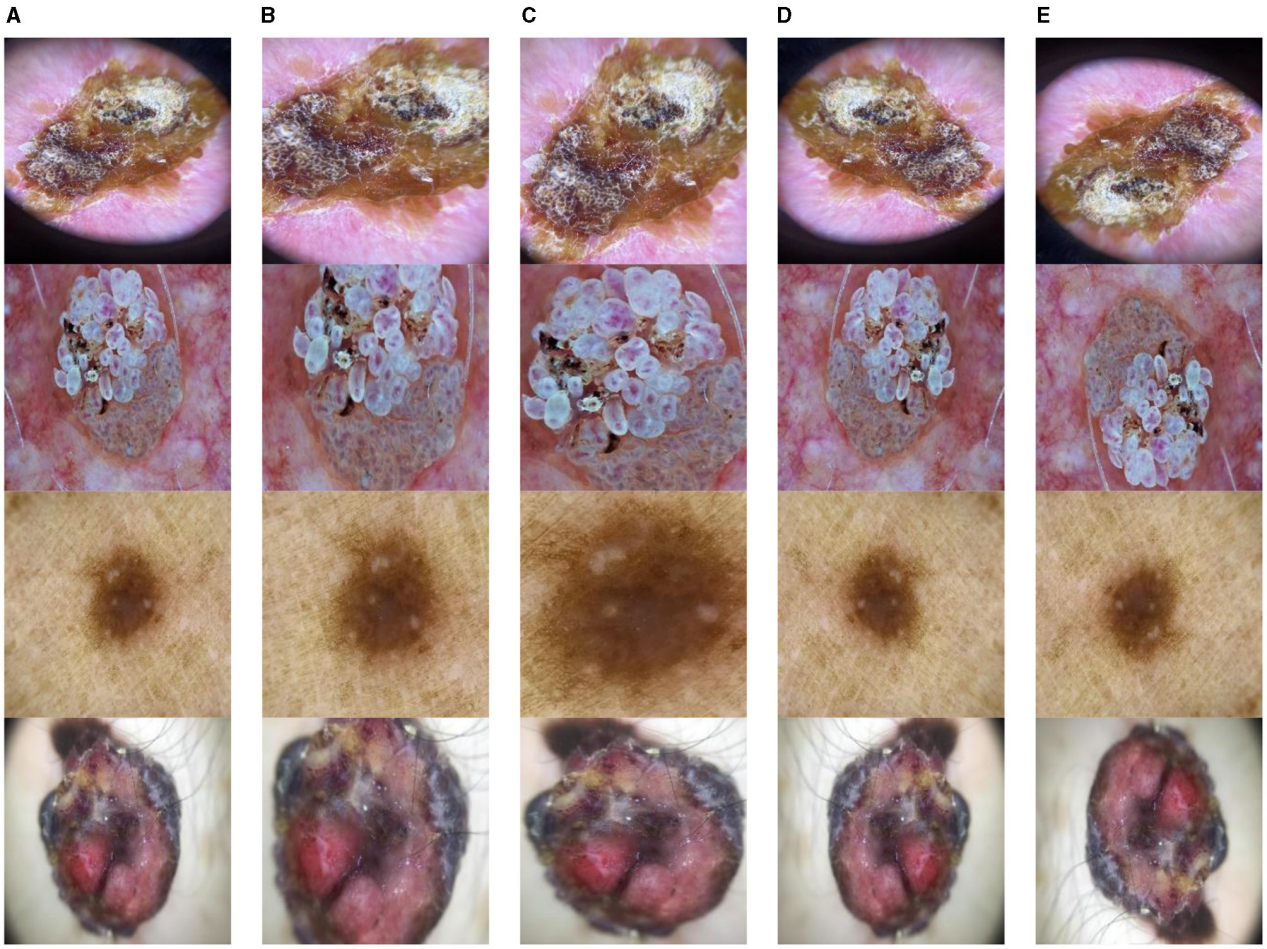
Data augmentation **(A)** original image, **(B)** rotate, **(C)** zoom, **(D)** horizontal flip, and **(E)** vertical flip.

Data Augmentation is modifying existing training data to generate new, synthetic training data. To enhance the volume of data available for training a network without collecting extra data, this is frequently employed in ML and DL (31). Data Augmentation provides various advantages, including improved model performance, reduced overfitting, robustness of models, and increased diversity. Data augmentation was performed on the ISIC datasets to improve their diversity. This research study diversifies the dataset by using rotate, zoom, horizontal flip, and vertical flip. This improves the training process of CNN models and enhances their performance. After augmentation, the datasets were split to form sets for training and testing the proposed CNN models. Some examples of the data augmentation techniques utilized in this research study are displayed in Figure 5.

### 3.3 Proposed CNN models

CNN is a popular DL model. A typical CNN architecture consists of two steps: feature extraction and classification. The CNN model extracts and varies features through five layers: the input, convolution, pooling, fully connected, and classification layers. CNN performs feature extraction and classification by deploying increasingly trainable layers stacked on each other. In the feature extraction phase of a CNN, convolutional and pooling layers are utilized, whereas fully connected classification layers are used in the classification phase. This paper proposed a system of three CNN models for three distinct classification tasks. The grid search technique was used to optimize the hyperparameters of each CNN model.

#### 3.3.1 Proposed CNN model 1 for skin lesion detection

**Figure 6:**
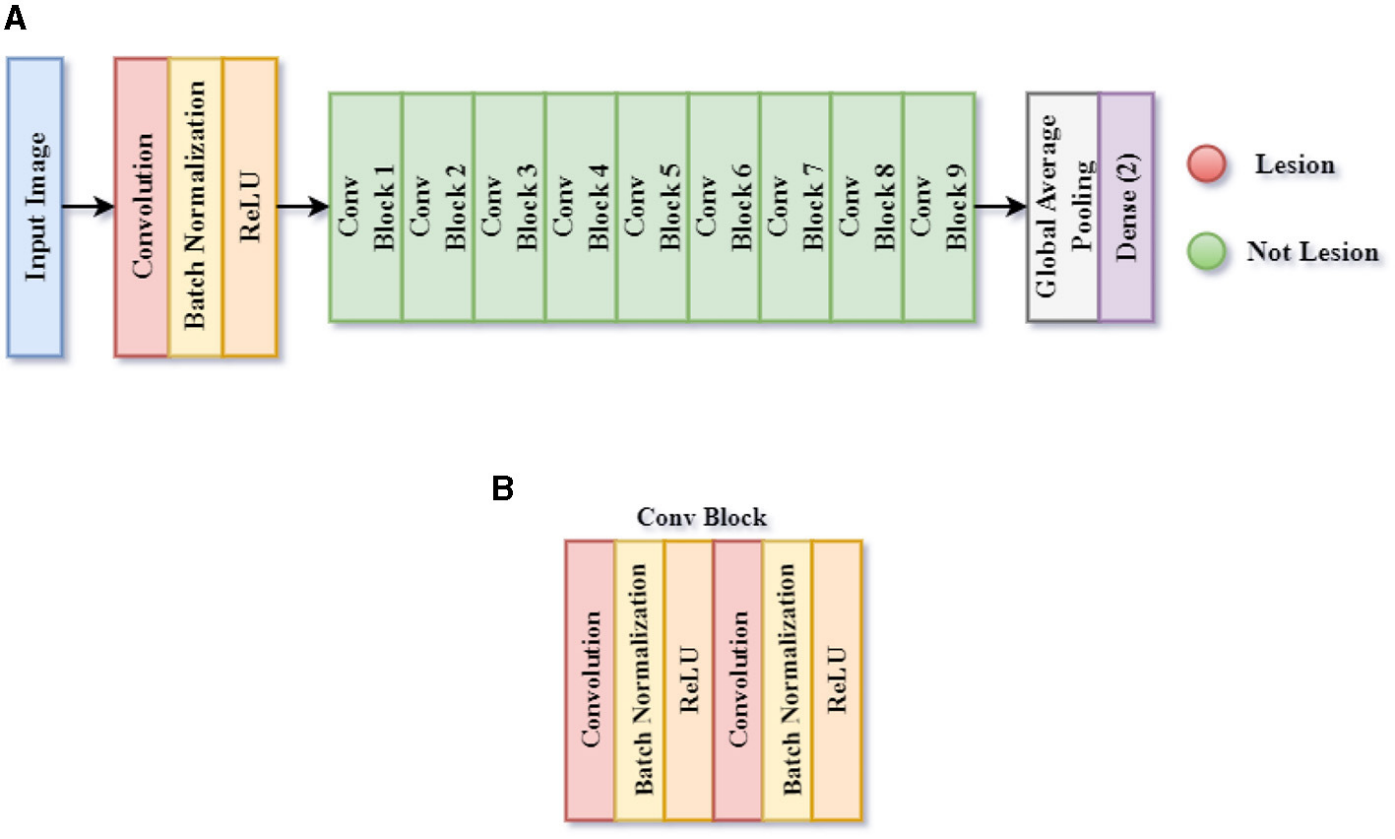
Framework of proposed CNN model 1 for skin lesion detection task 1. **(A)** CNN model 1, and **(B)** Conv block.

The first CNN model determines whether a patient's skin image contains a skin lesion, as it is designed to detect skin lesions. This classification is referred to throughout this article as Classification Task 1. Figure 6 illustrates the structure of the proposed CNN architecture 1, which includes 60 layers: 1 Input, 19 Convolutions, 19 ReLU, 19 Batch Normalization, 1 Global Average Pooling, and 1 Classification layer. The output layer consists of two neurons because the initial CNN architecture aims to classify an image into two categories. The SoftMax activation function uses the dense layer's input, a 2-D feature vector, to determine the presence or absence of a lesion.

Table 2 shows the model summary of the first CNN architecture. The model summary details the input image size, output image size, and the parameters of 1 Input Layer, 10 Convolutional Blocks, 1 Global Average Pooling, and 1 Dense layer. Convolutional Blocks 1–9 consist of 6 layers each: 1 Conv2D layer, 1 Depthwise Conv2D layer, 2 Batch Normalization layers, and 2 activation layers. Convolutional Block 10 consists of 3 layers: 1 Conv2D, 2 Batch Normalization layers, and 2 activation layers. The model consists of a total of 2,147,522 parameters. The parameters are split into trainable and non-trainable categories consisting of 2,133,826 parameters and 13,696 parameters, respectively.

**Table 2:** Model summary of proposed CNN model 1 for skin lesion detection.

**Layer name**	**Input image size**	**Output image size**	**Number of parameters**
Input layer	-	224 × 224 × 3	0
Convolutional block 1	224 × 224 × 3	112 × 112 × 32	1,408
Convolutional block 2	112 × 112 × 32	56 × 56 × 64	3,136
Convolutional block 3	56 × 56 × 64	56 × 56 × 128	10,368
Convolutional block 4	56 × 56 × 128	28 × 28 × 128	18,560
Convolutional block 5	28 × 28 × 128	28 × 28 × 256	37,120
Convolutional block 6	28 × 28 × 256	14 × 14 × 256	69,888
Convolutional block 7	14 × 14 × 256	14 × 14 × 512	139,776
Convolutional block 8	14 × 14 × 512	14 × 14 × 512	270,848
Convolutional block 9	14 × 14 × 512	14 × 14 × 1,024	541,696
Convolutional block 10	14 × 14 × 1,024	14 × 14 × 1,024	1,052,672
Global average pooling	14 × 14 × 1,024	1,024	0
Dense	1,024	2	2,050

#### 3.3.2 Proposed CNN model 2 for benign/malignant classification of skin lesions

**Figure 7:**
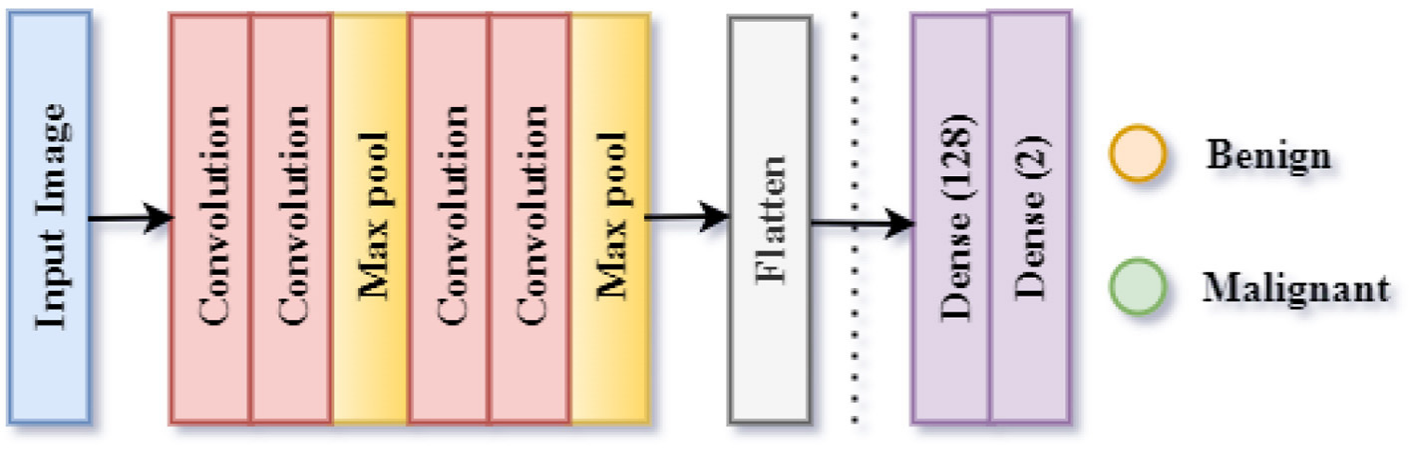
Framework of proposed CNN model 2 for benign/malignant lesion classification task 2.

The lesions can also be classified separately into Benign or Malignant. The third CNN model is used for the implementation of this classification. This classification is referred to as Classification Task 3 throughout the paper. As illustrated in Figure 7, the proposed CNN design for Classification Task 2 is comprised of 10 weighted layers: 1 Input, 4 Convolutional layers, 2 Max Pooling layers, 1 Dense layer, 1 Dropout layer, and 1 classification layer. As the CNN 2 model is simulated for the classification of an image into two classes, the output layer contains two nodes. The SoftMax activation function predicts the final lesion type after receiving a 2-D feature vector as input from the final dense layer.

The model summary of the second CNN design is highlighted in Table 3. The model summary provides information regarding the input image size, output image size, and parameters for 4 Conv2D layers, 2 MaxPooling layers, 1 Flatten layer, and 2 Dense layers. There are 2,881,314 parameters in the architecture. There are no non-trainable parameters, as every parameter is trainable.

**Table 3:** Model summary of proposed CNN model 2 for benign/malignant classification of skin lesions.

**Layer name**	**Input image size**	**Output image size**	**Number of parameters**
Input layer	-	222 × 222 × 3	0
Conv2D	222 × 222 × 3	222 × 222 × 16	448
Conv2D	222 × 222 × 16	220 × 220 × 16	2,320
MaxPooling2D	220 × 220 × 16	110 × 110 × 16	0
Conv2D	110 × 110 × 16	108 × 108 × 8	1,160
Conv2D	108 × 108 × 8	106 × 106 × 8	584
MaxPooling2D	106 × 106 × 8	53 × 53 × 8	0
Flatten	53 × 53 × 8	22,472	0
Dropout	22,472	22,472	0
Dense	22,472	128	2,876,544
Dense	128	2	258

#### 3.3.3 Proposed CNN model 3 for classification of benign/malignant skin lesion in seven classes

**Figure 8:**
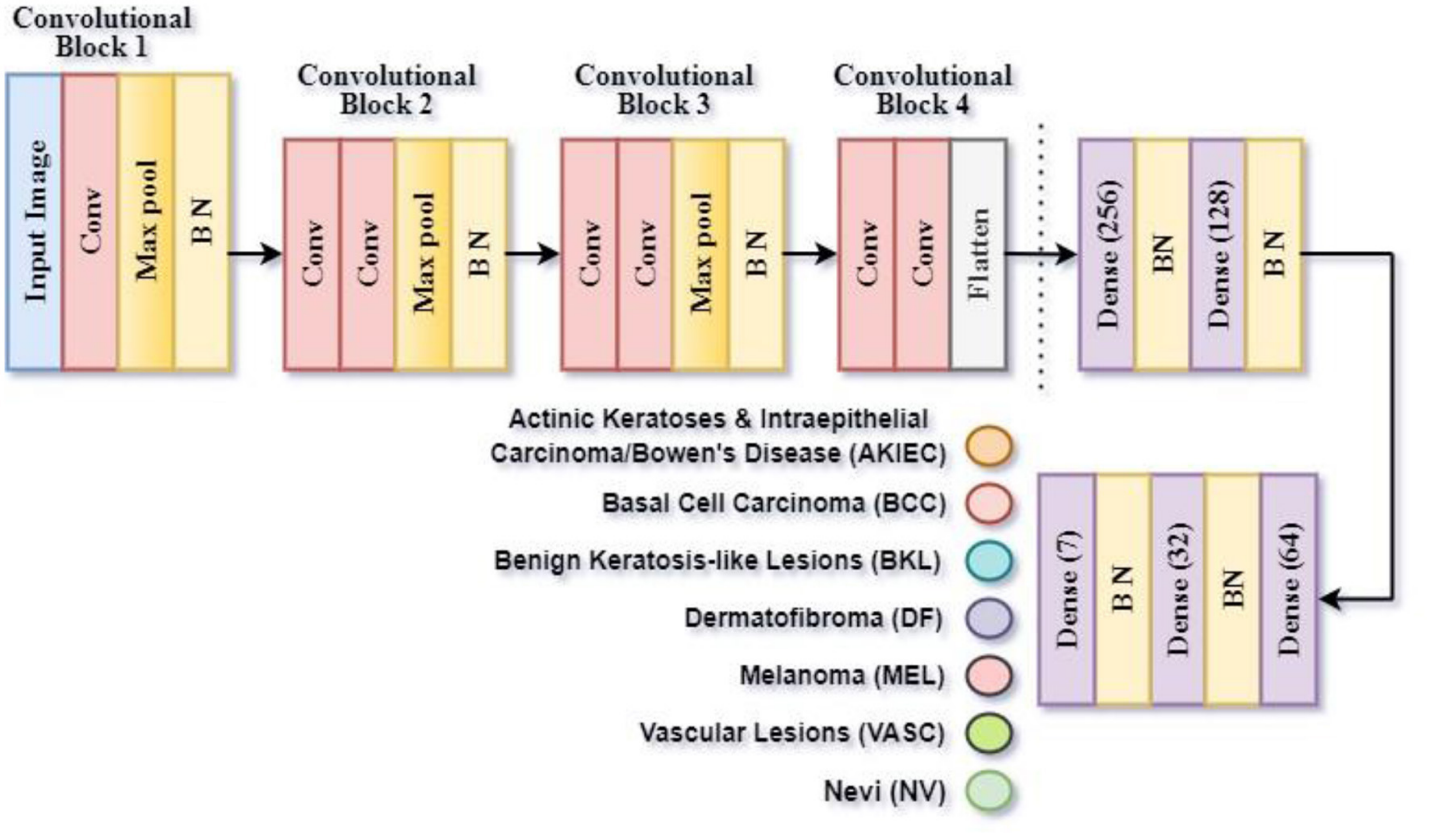
Framework of proposed CNN model 3 for classification task 3 of benign/malignant skin lesion classification in seven classes.

The third CNN model is implemented for the classification of images into seven classes: AKIEC, BCC, BKL, DF, MEL, NV, and VASC. This classification is referred to as Classification Task 3 throughout the article. As shown in Figure 8, the proposed CNN design to Classify Task 3 consists of 24 weighted layers: 1 Input, 7 Convolutional layers, 7 Batch Normalization layers, 3 Max Pooling layers, 4 Dense layers, 1 Dropout layer, and 1 Classification layer. The output layer includes seven neurons since the third CNN design is intended to classify an image into seven classes. The SoftMax classifier creates the final lesion type prediction, which receives an input of a seven-dimensional feature vector from the last dense layer.

Table 4 presents the model summary of the third CNN design. The model summary details the input image size, output image size, and the parameters of 1 Input Layer, 4 Convolutional Blocks, 1 Flatten, 1 Dropout, 4 Batch Normalization, and 5 Dense layers. Convolutional Block 1 consists of 3 layers: 1 Conv2D, 1 MaxPooling2D, and 1 Batch Normalization layer. Convolutional Blocks 2 and 3 consist of 4 layers: 2 Conv2D, 1 MaxPooling2D, and 1 Batch Normalization layers. Convolutional Block 4 consists of 3 layers: 2 Conv2D and 1 MaxPooling2D layers. The model consists of a total of 1,275,079 parameters. The parameters are split into trainable and non-trainable categories consisting of 1,273,671 parameters and 1,408 parameters, respectively.

**Table 4:** Model summary of proposed CNN model 3 for classification of benign/malignant skin lesion in seven classes.

**Layer Name**	**Input Image Size**	**Output Image Size**	**Number of Parameters**
Input layer	-	28 × 28 × 3	0
Convolutional block 1	28 × 28 × 3	14 × 14 × 32	1,024
Convolutional block 2	14 × 14 × 32	7 × 7 × 64	55,680
Convolutional block 3	7 × 7 × 64	3 × 3 × 128	221,952
Convolutional block 4	3 × 3 × 128	1 × 1 × 256	885,248
Flatten	1 × 1 × 256	256	0
Dropout	256	256	0
Dense + batch normalization 1	256	256	66,816
Dense + batch normalization 2	256	128	33,408
Dense + batch normalization 3	128	64	8,512
Dense + batch normalization 4	64	32	2,208
Dense	32	7	231

## 4 Experimental setup

Several obstacles have developed in the utilization of CNNs as their application in the discipline of medical imaging analysis has grown. More significant computational expenses are generated when the designs, which are improved to produce more effective outcomes, become deeper and the input images become of better superiority. Utilizing robust hardware and tuning the hyper-parameters of the existing models are crucial for lowering these computing costs and producing superior outcomes. As a result, the suggested CNN models virtually all have their key hyper-parameters automatically adjusted using the grid search optimization approach. When the search space for the value range is limited, the grid search optimization method is a useful alternative to CNN hyper-parameter optimizations. Grid Search Optimization was therefore implemented in this research study for each classification task for optimizing the hyper-parameters of each of the suggested CNN architectures.

Furthermore, to scientifically validate the study's findings, analyzing the classification parameters to classify image research is essential. If not done properly, then the performance of the classification research remains without evidence and is thus academically insufficient. The performance of each proposed CNN model for the specified classification tasks of skin lesions was evaluated using several methods, such as the Loss Analysis Plot, Accuracy Analysis Plot, and Confusion Matrix.

### 4.1 Hyperparameter optimization using grid search

To identify the ideal set of hyperparameters for proposed CNN models, the Grid Search Optimisation method has been used for hyperparameter optimization. Values for hyperparameters are predetermined prior to the beginning of the process of learning as they cannot be inferred solely from the data (32). Architectures for CNN models are relatively complex and contain many hyperparameters. To enhance the performance of proposed models, two types of hyperparameters are tuned, i.e., Architectural hyperparameters and fine modification hyperparameters. Architectural hyper-parameters include the convolutional layers, pooling layers, fully connected layers, and the activation function. In contrast, Batch size and learning rate, conversely, are referred to as acceptable alterations of hyper-parameters. In grid search, a grid of potential results for the hyperparameters mentioned above is first defined, and the CNN model is then trained with all feasible combinations to ascertain which combination produces the greatest performance.

The stages involved in grid search optimization for CNN models are as follows:

1. Hyperparameter grid formation: for each hyperparameter that is to be optimized, a range of possible values is set.
2. Potential combination generation: all potential combinations of hyperparameters are generated from the range of values in the formed grid.
3. Model evaluation: the proposed model is implemented using each potential combination of the hyperparameters, and its performance is evaluated.
4. Determination of optimized hyperparameter combination: the hyperparameter combination with the best results is determined.
5. Utilization of optimized hyperparameters: the proposed design is retrained and implemented with the optimized hyperparameters derived from the grid search.

The Grid Search Optimization for each classification task has been shown in Tables 5–**7**. Table 5 shows the optimized hyperparameters derived from the grid search of the first proposed CNN model implemented for the detection of Skin Lesions.

**Table 5:** Optimum hyper-parameters results achieved by grid search of proposed CNN model 1 for skin lesion detection task.

**Hyper parameters**	**Hyper parameter range**	**Optimized value**
Convolution layers	[13–19]	19
Global average pooling layer	[1–4]	1
Fully connected layers	[1–4]	1
Activation function	[ReLU, Softmax, Sigmoid, Leaky ReLU]	ReLU, Softmax
Batch size	[16, 64, 128]	64
Learning rate	[0.0001, 0.01, 0.001, 0.00001]	0.01
Number of epochs	[10, 20, 30, 40, 50]	30

Table 6 shows the optimized hyperparameters derived from the grid search of the second proposed CNN model implemented for the classification of Skin Lesions as Benign or Malignant.

**Table 6:** Optimum hyper-parameters results achieved by grid search of proposed CNN model 2 for benign/malignant classification of skin lesions.

**Hyper parameters**	**Hyper parameter range**	**Optimized value**
Convolution layers	[1–4]	4
Max pooling layers	[1–4]	2
Fully Connected layers	[1–4]	2
Activation function	[ReLU, Softmax, Sigmoid, Leaky ReLU]	ReLU, Sigmoid
Batch size	[16, 64, 128]	64
Learning rate	[0.0001, 0.01, 0.001, 0.00001]	0.001
Number of epochs	[10, 20, 30, 40, 50]	30

Table 7 shows the optimized hyperparameters derived from a grid search of the third proposed CNN model implemented for the Classification of Benign/Malignant Skin Lesions in seven distinct classes.

**Table 7:** Optimum hyper-parameters results achieved by grid search of proposed CNN model 3 for classification of benign/malignant skin lesions in seven classes.

**Hyper parameters**	**Hyper parameter range**	**Optimized value**
Convolution layers	[3–9]	7
Max pooling layers	[1–4]	3
Fully connected layers	[2–5]	5
Activation function	[ReLU, Softmax, Sigmoid, Leaky ReLU]	ReLU, Softmax
Batch size	[16, 64, 128]	64
Learning rate	[0.0001, 0.01, 0.001, 0.00001]	0.001
Number of epochs	[10, 20, 30, 40, 50]	30

The optimized values of hyperparameters derived from the grid search algorithm are finally used to simulate and evaluate the CNN models for different categorization tasks.

### 4.2 Results

Analyzing the performance of classification research is essential to validate the study's findings scientifically. If not done properly, then the performance of the classification research remains without evidence and is thus academically insufficient. This research evaluates the performance of the CNN models implemented for the three Classification Tasks using Analysis Plots of Loss and Accuracy and Confusion Matrices. These give an overall summary of the performance of the CNN models by providing information regarding learning rate and overfitting during training and performance parameters such as Accuracy, Precision, Recall, and F1 Score during the model implementation on the test sets.

The Loss and Accuracy Analysis Plots are used to determine several parameters observed during the training of the CNN models. The Loss Analysis Plot highlights the loss of a model during the training and validation phase. It is used to observe whether the model had a good learning rate. The Accuracy Analysis Plot highlights the accuracy of a model during the training and validation phase. The gap between the training accuracy plot and validation accuracy plot represents whether a problem of overfitting had occurred.

A table used to assess the efficiency of a classification design is referred to as a confusion matrix or error matrix. It is a multi-dimensional matrix that displays the actual and predicted class labels for each piece of data in a classification task's summary results.

#### 4.2.1 Performance of CNN model 1 for skin lesion detection

**Figure 9:**
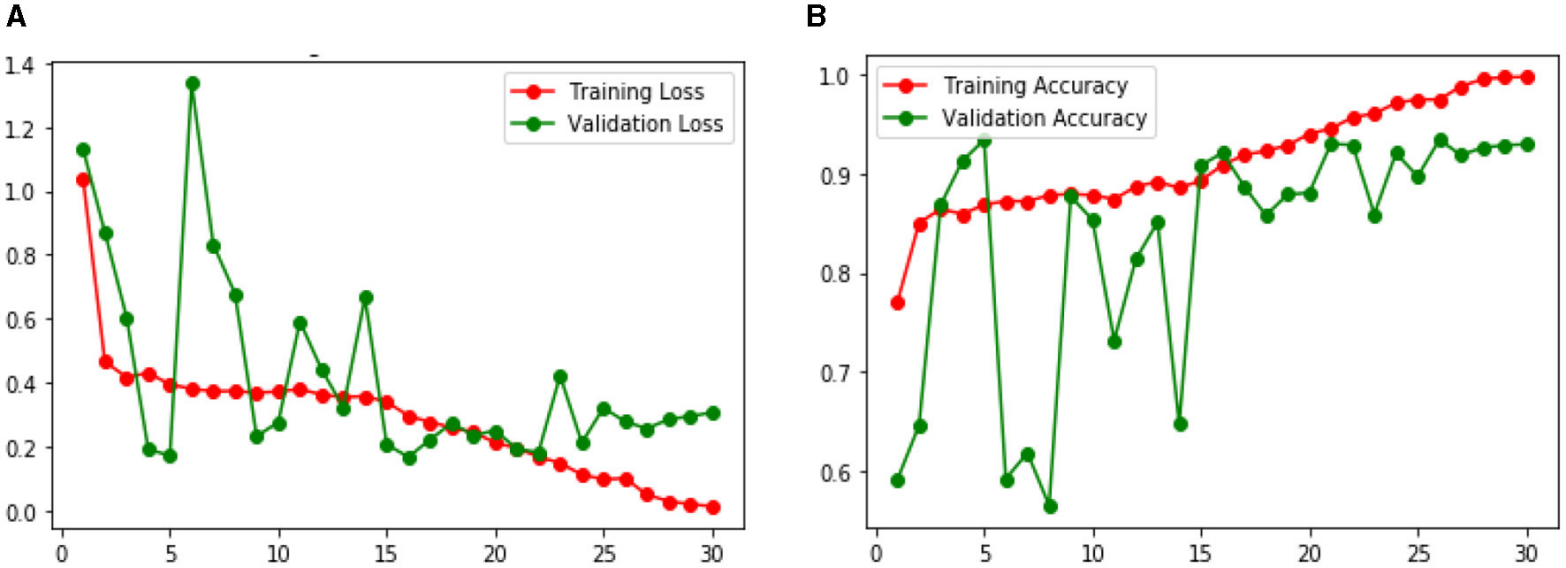
Results of proposed CNN model 1 for classification task 1 **(A)** Loss analysis plot, and **(B)** accuracy analysis plot.

Figure 9 shows the Loss and Accuracy Analysis Plots obtained by the first CNN model for Classification Task 1. Figure 9A highlights the loss incurred by the proposed CNN model during the training and validation phase. The training loss was 0.10, and the validation loss was observed to be 0.28. It can be seen that since the slope of the training and validation plots is exponentially decreasing, the model had a good learning rate. Figure 9B highlights the accuracy obtained by the proposed CNN model for the training and validation phase. The training accuracy achieved by the design was observed as 0.98, and the validation accuracy was observed as 0.93. Since the gap between the training and validation accuracy is low, negligible overfitting in the model is represented.

**Figure 10:**
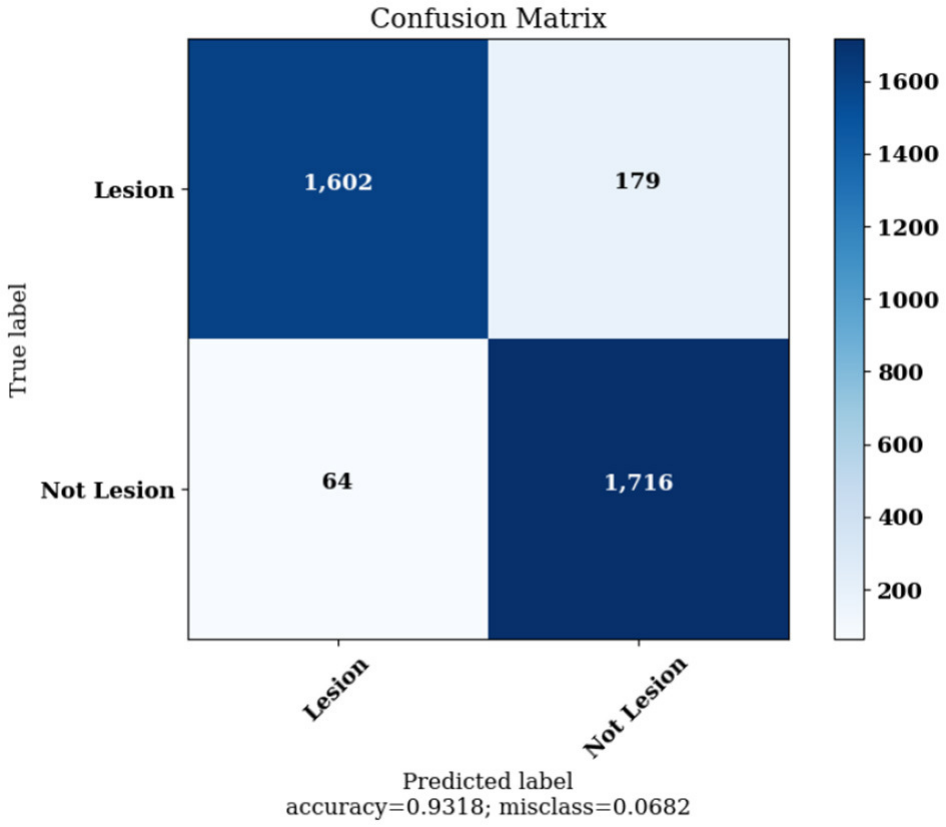
Confusion matrix achieved for classification task 1.

Figure 10 highlights the Confusion Matrix the CNN for Classification Task 1 formed. For classification task 1, the confusion matrix is a two-dimensional matrix that indicates the predictions made by the model for classifying images into two classes, detecting whether the image contains skin lesions or not.

#### 4.2.2 Performance of CNN model 2 for benign/malignant classification of skin lesions

**Figure 11:**
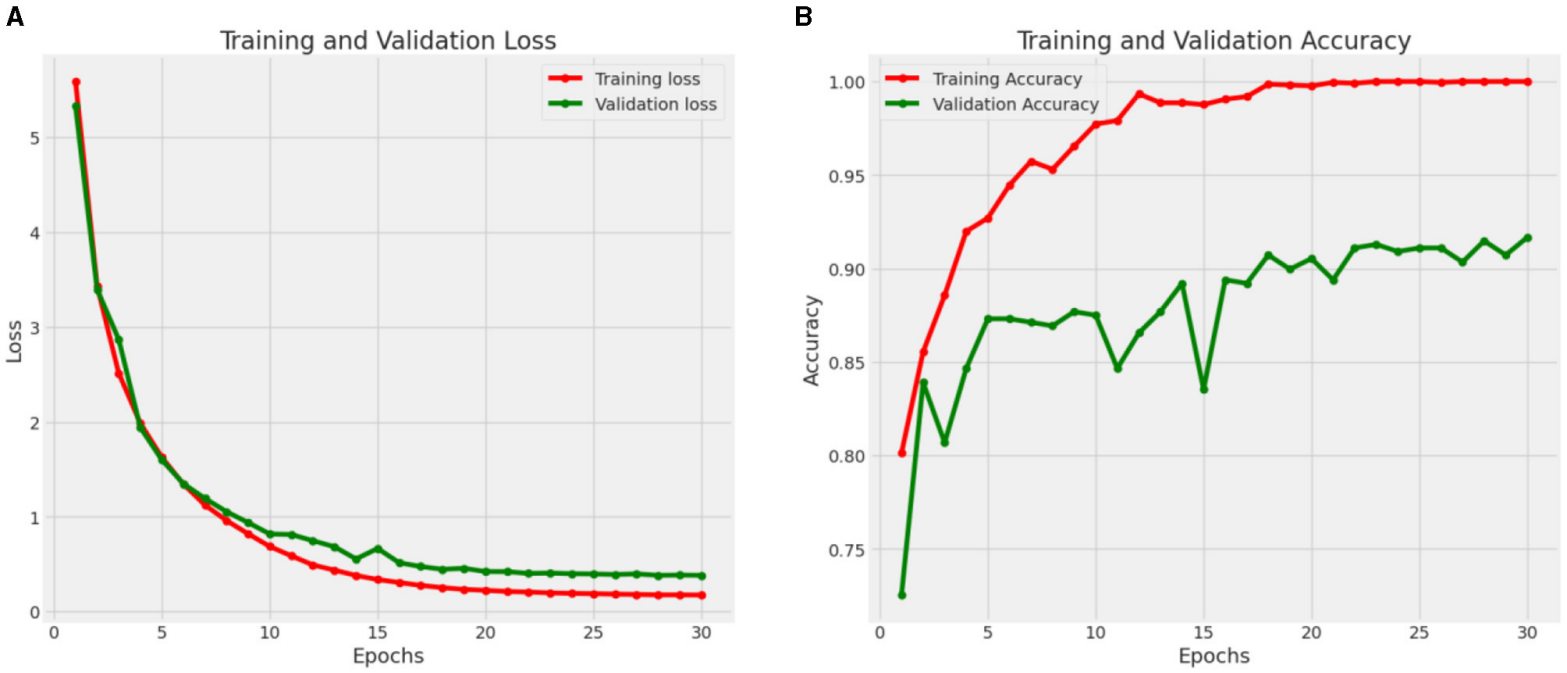
Results of proposed CNN model 2 for classification task 2. **(A)** Loss analysis plot, and **(B)** accuracy analysis plot.

Figure 11 shows the Loss and Accuracy Analysis Plots obtained by the second CNN model for Classification Task 2. Figure 11A highlights the loss incurred by the proposed CNN model during the training and validation phase. The training loss was 0.10, and the validation loss was 0.21. It can be observed that since the slope of the training and validation plots is exponentially decreasing, the model had a good learning rate. Figure 11B highlights the accuracy obtained by the proposed CNN model during the training and validation phase. The training accuracy achieved by the model was observed as 0.98, and the validation accuracy was observed as 0.92. Since the gap between the training and validation accuracy is low, negligible overfitting in the model is represented.

**Figure 12:**
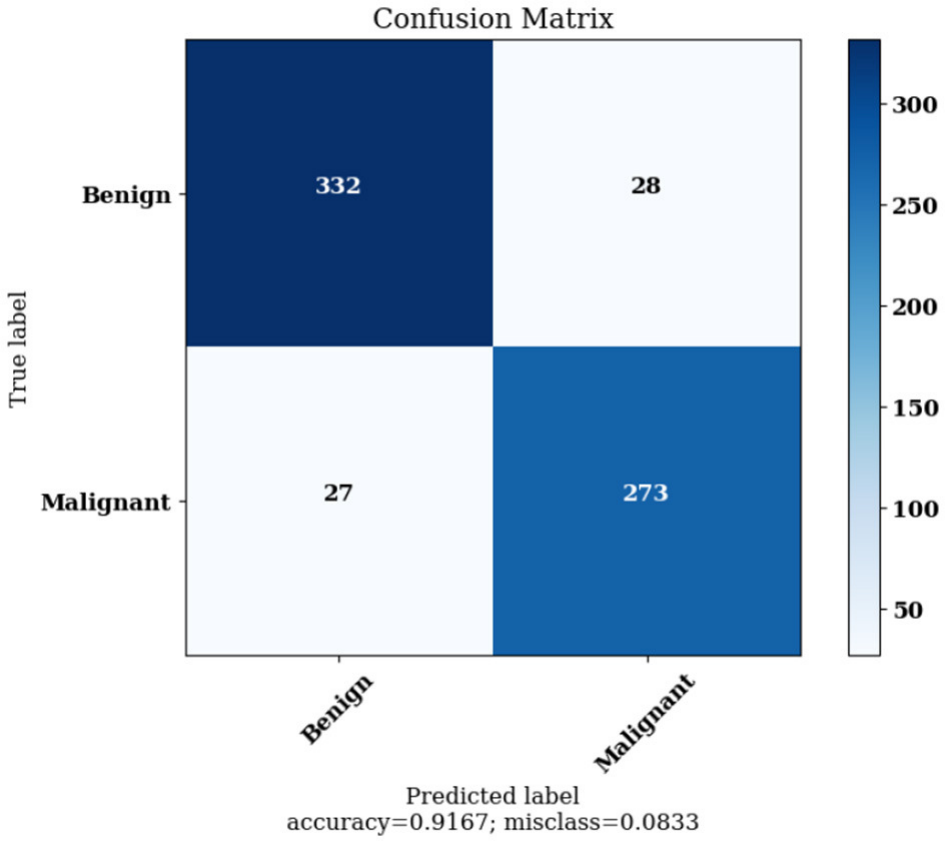
Confusion matrix achieved for classification task 2.

Figure 12 highlights the Confusion Matrix the CNN for Classification Task 2 formed. For classification task 2, the confusion matrix is a two-dimensional matrix that indicates the predictions made by the model for classifying images into two classes, showing whether the lesion detected is benign or malignant.

#### 4.2.3 Performance of CNN model 3 for classification benign/malignant skin lesions in seven classes

**Figure 13:**
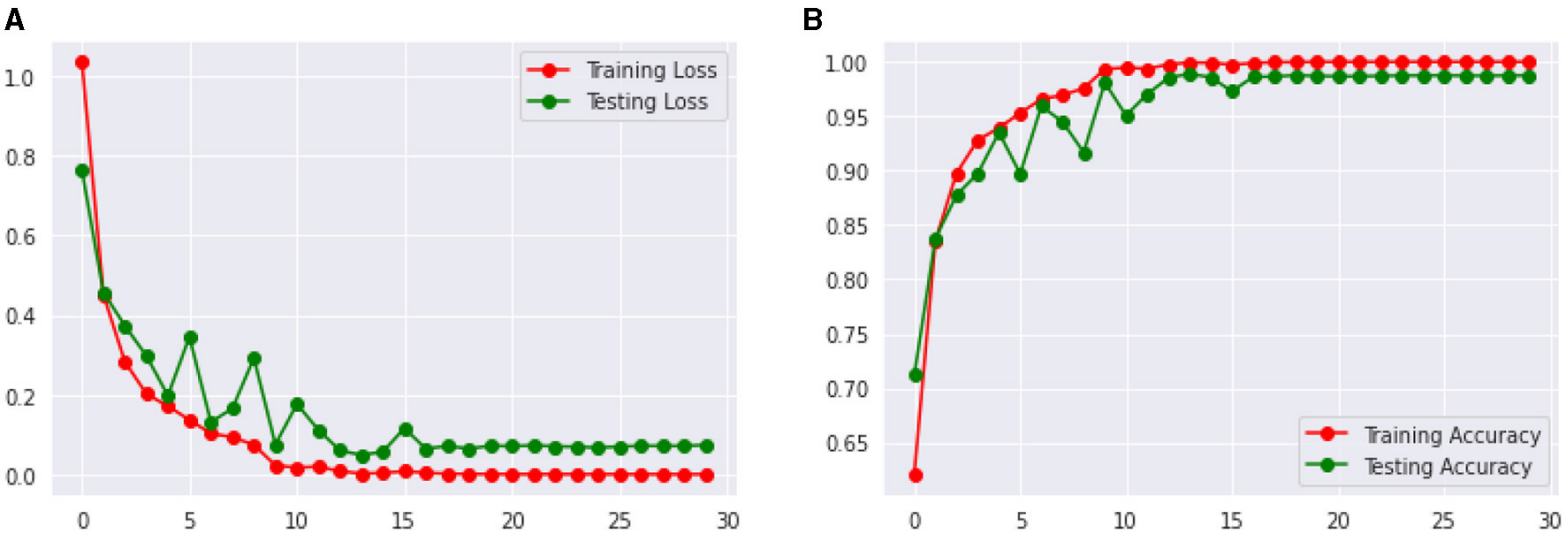
Results of proposed CNN model 3 for classification task 3. **(A)** Loss analysis plot, and **(B)** accuracy analysis plot.

**Figure 14:**
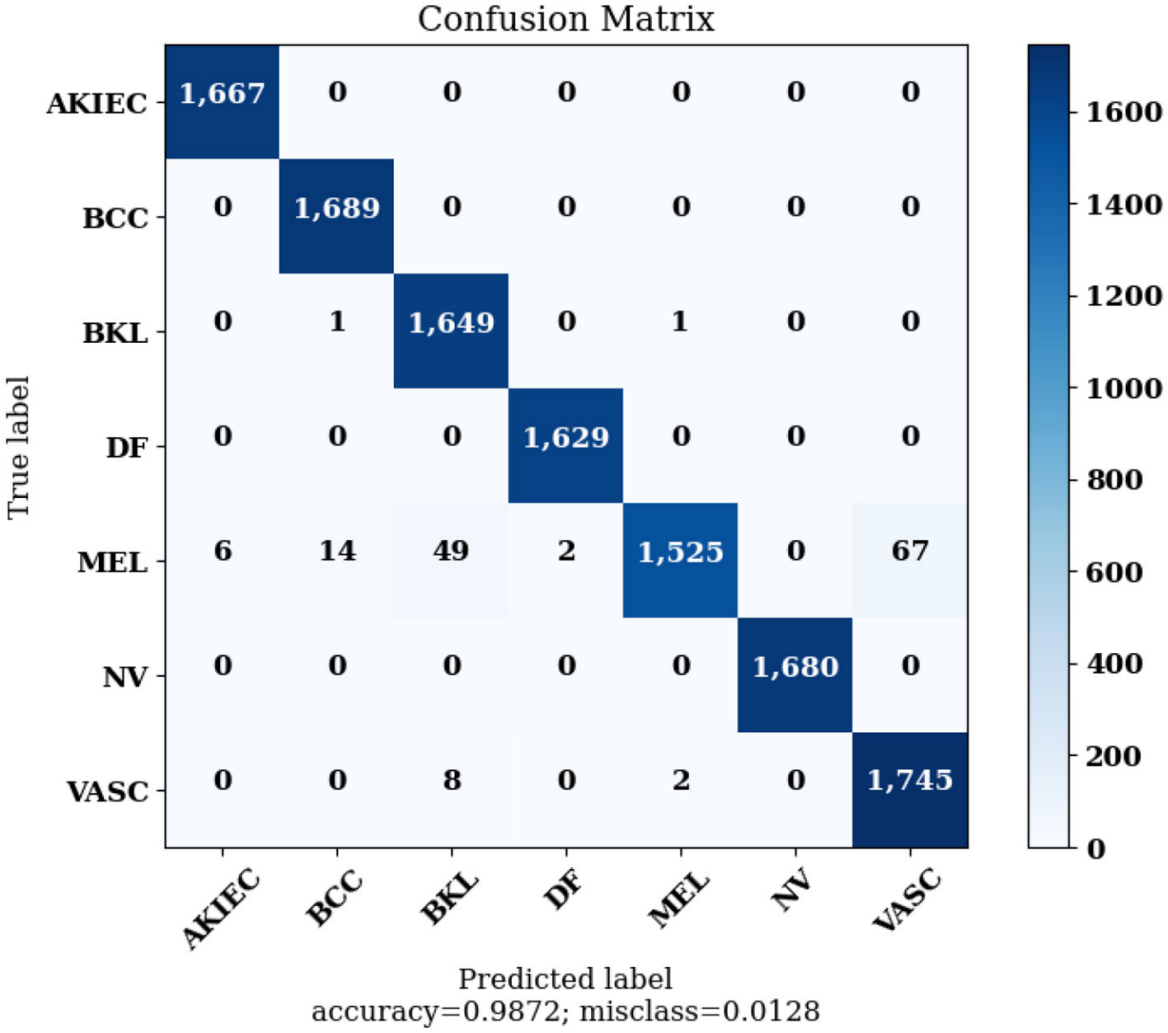
Confusion matrix achieved for classification task 3.

Figure 14 shows the Loss and Accuracy Analysis Plots obtained by the third CNN model for Classification Task 3. Figure 13A highlights the loss incurred by the proposed CNN model during the training and validation phase. The training loss was 0.07, and the validation loss was 0.11. It can be seen that since the slope of the training and validation plots is exponentially decreasing, the model had a good learning rate. Figure 13B highlights the accuracy obtained by the proposed CNN model during the training and validation phase. The training accuracy achieved by the model was observed as 0.99, and the validation accuracy was observed as 0.98. Since the gap between the training and validation accuracy is low, negligible overfitting in the model is represented.

Figure 14 highlights the Confusion Matrix the CNN for Classification Task 3 formed. For classification task 3, the confusion matrix is a multi-dimensional matrix that indicates the predictions made by the model for the classification of images into seven classes according to the type of lesion detected. The scale of 0 to 6 on the x-axis and y-axis represents the classes for classification task 3, which are as follows: 0 for AKIEC, 1 for BCC, 2 for BKL, 3 for DF, 4 for MEL, 5 for NV, and 6 for VASC.

The Confusion Matrices displayed in Figures 10, 12, 14 are utilized to analyze specific metrics for each CNN model implemented for the classification tasks. Table 8 represents Confusion Matrix values for each class of each classification task and the evaluated performance metrics, including Precision, Recall, F1 Score, and Accuracy.

**Table 8:** Performance metrics for detection and classification of skin lesions.

**CNN Model**	**Classes**	**TP**	**TN**	**FP**	**FN**	**Precision**	**Recall/Sensitivity**	**Specificity**	**F1 Score**	**Accuracy**
CNN Model 1	Lesion	1,602	1,716	64	179	0.96	0.91	0.96	0.93	93.18%
	Not Lesion	1,716	1,602	179	64	0.90	0.96	0.90	0.93	
CNN Model 2	Benign	332	273	27	28	0.93	0.92	0.91	0.92	91.67%
	Malignant	273	332	28	27	0.90	0.91	0.92	0.90	
CNN Model 3	AKIEC	1,667	9,917	6	0	1	1	0.99	1	98.72%
	BCC	1,689	9,895	15	0	0.99	1	1	1	
	BKL	1,649	9,935	49	2	0.98	0.99	0.99	0.99	
	DF	1,629	9,955	2	0	1	1	1	1	
	MEL	1,525	1,0059	3	138	0.99	0.92	1	0.95	
	NV	1,680	9,904	0	0	1	1	1	1	
	VASC	1,745	9,839	67	10	0.95	0.99	0.99	0.97	

As seen from Table 8, each of the CNN models achieved excellent performance. CNN model 1 simulated the detection of skin lesions and achieved an accuracy of 93.18%. CNN model 2 for the Benign/Malignant Skin Lesions classification attained an accuracy of 91.67%. CNN model 3 for Classification of Benign/Malignant Skin diseases in Seven Classes achieved an accuracy of 98.72%.

#### 4.2.4 Comparative result analysis of hyperparameter optimisation using grid search

To validate the implementation of the Hyperparameter Optimisation using the Grid Search technique employed in this study, Table 9 presents a comparative analysis of the results obtained for the three classification tasks by the CNN models without and with the implementation of the Grid Search technique. A comparison of the aggregate of the performance metrics Precision, Recall, Specificity, F1 Score, and Accuracy is presented for each classification task.

**Table 9:** Comparison of results for hyperparameter optimization using grid search.

	**Without hyperparameter optimisation**					**Hyperparameter optimisation using grid search**				
**CNN model**	**Precision**	**Recall**	**Specificity**	**F1 Score**	**Accuracy**	**Precision**	**Recall**	**Specificity**	**F1 Score**	**Accuracy**
CNN model 1	0.88	0.86	0.86	0.87	86.73%	0.93	0.94	0.93	0.93	93.18%
CNN model 2	0.82	0.84	0.81	0.83	83.42%	0.91	0.92	0.92	0.91	91.67%
CNN model 3	0.85	0.84	0.83	0.85	85.61%	0.99	0.98	1	0.99	98.72%

As observed from Table 9, using Grid Search for Hyperparameter Optimisation leads to significantly better results throughout all performance metrics when compared to no implementation of hyperparameter optimization. Using Grid Search leads to consistently high performance metrics thus validating the performance of the models for each classification task further.

#### 4.2.5 Comparison of proposed work with related studies

Table 10 highlights the comparison of the proposed work in this research study. The various studies are compared based on several categories, including Classification Type, Dataset Utilized, Number of Images, Technique Implemented, and Accuracy Achieved.

**Table 10:** Comparison of proposed work with related studies.

**References**	**Classification type**	**Dataset utilized**	**Number of images**	**Technique implemented**	**Accuracy achieved**
Cassidy et al. (14)	AKIEC BCC MEL SCC	Internet	3,753	AlexNet	AKIEC = 92.3% BCC = 91.8% MEL = 94.2% SCC = 95.1%
Liang (15)	AKIEC BCC BKL NV MEL	HAM10000 ISIC Archive	11,444	CNN	p < 0.001
Dorj et al. (16)	MEL BCC	PH^2^ ISIC 2016 Challenge ISIC 2018 Challenge	7,849	Feature Fusion between AlexNet & VGG16	99%
Maron et al. (17)	MEL NV	HAM10000 ISIC Archive	804	ResNet50	75.03%
Polat and Koc (23)	AKIEC BCC BKL DF MEL NV VASC	HAM10000	10,015	CNN	86.5%
Duggani and Nath (24)	AKIEC BCC BKL DF MEL NV VASC	HAM10000	10,015	CNN	95.18%
Khan et al. (25)	Benign Malignant	Internet	3,297	VGG16	89.09%
Shetty et al. (26)	AKIEC BCC BKL DF MEL NV VASC	HAM10000	10,015	Xception	96.40%
Anand et al. (27)	AKIEC BCC BKL DF MEL NV VASC	HAM10000	10,015	Lightweight Dynamic Kernel CNN	97.85%
Anand et al. (28)	AKIEC BCC BKL DF MEL NV VASC SCC	ISIC 2019 Challenge	25,331	XAI	94.47%
Proposed Work	Lesion Not lesion, Benign Malignant, AKIEC BCC BKL DF MEL NV VASC	ISIC Archive	17,806 3,297 46,935	CNN Model 1 CNN Model 2 CNN Model 3	93.18% 91.67% 98.72%

## 5 Conclusion and future work

Modern advancements in deep learning have led to the expansion of machine learning research and study beyond feature engineering to architectural engineering. This study presents a system of CNN models for comprehensive skin lesion diagnosis. Three robust CNN architectures were presented for three skin lesion classification tasks involving the classification of a skin lesion, determining whether a lesion is benign or malignant, and classifying the skin lesion by type. Annotated images from the ISIC Archive were utilized to form three distinct datasets for each classification task. For each task, the datasets contained various images according to the classification tasks. Grid Search optimization was implemented in each of the proposed CNN models to optimize the hyperparameters and obtain the best results. The detection of skin lesions was performed with an accuracy of 93.18 percent. In addition, the classification of skin lesions based on whether they were benign or malignant was obtained with an impressive 91.67 percent accuracy. The classification of cutaneous lesions into seven distinct categories was accomplished with a high degree of precision (98.72%). The results and performance of the proposed CNN models demonstrate the effectiveness of deep-learning approaches for Skin lesion classification. This research study proposes CNN models that can be used to aid dermatologists with initial skin lesion classification screening. Although the primary emphasis of the study was on CNN models, it is suggested that future research should consider investigating more sophisticated models, such as Transformers or hybrid architectures that integrate CNNs with Recurrent Neural Networks (RNNs) or attention techniques. The designs mentioned above have shown potential in several fields and might potentially enhance the precision and resilience of skin lesion data categorization. The integration of other data sources, such as histopathology pictures, patient medical history, or genetic information, has the potential to augment the efficacy of the model by offering a comprehensive perspective on the patient's medical state. The use of a multimodal approach has the potential to enhance the precision and customization of diagnostic instruments. Future research endeavors may prioritize the adaptation of these models to facilitate their real-time implementation inside clinical environments. Potential areas of focus may include the creation of interfaces that are intuitive and easy to use for dermatologists, as well as the incorporation of pre-existing medical imaging technologies. The validation of the efficacy of these models in real-world contexts via the implementation of clinical trials is crucial for the successful shift from research to practical application. Future research endeavors may prioritize the adaptation of these models to facilitate their real-time implementation inside clinical environments. Potential areas of focus may include the creation of interfaces that are intuitive and easy to use for dermatologists, as well as the incorporation of pre-existing medical imaging technologies. The validation of the efficacy of these models in real-world contexts via the implementation of clinical trials is crucial for the successful shift from research to practical application. The use of explainability approaches such as Grad-CAM or SHAP has the potential to improve the interpretability of CNN models, hence enhancing their reliability and facilitating their integration into clinical practice. Implementing this approach would enable healthcare practitioners to comprehend the underlying rationale behind the model's predictions, hence enhancing their trust in the outcomes.

## Data Availability

The raw data supporting the conclusions of this article will be made available by the authors, without undue reservation.

## References

[1] McLafferty E. The integumentary system: anatomy, physiology and function of skin. Nurs Stand. (2012) 27:35–42. 10.7748/ns2012.09.27.3.35.c9299 28080826

[2] Romanovsky A. Skin temperature: its role in thermoregulation. Acta Physiol (Oxf). (2014) 210:498–507. 10.1111/apha.12231 24716231 PMC4159593

[3] Dhivyaa CR Sangeetha K Balamurugan M Amaran S Vetriselvi T Johnpaul P. et al. Skin lesion classification using decision trees and random forest algorithms. J Ambient Intell Hum. Comput. 10.1007/s12652-020-02675-8

[4] Yilmaz E Trocan M. Benign and Malignant Skin Lesion Classification Comparison for Three Deep-Learning Architectures. In: Nguyen N Jearanaitanakij K Selamat A Trawiński B Chittayasothorn S, editor. Intelligent Information and Database Systems. ACIIDS 2020 Lecture Notes in Computer Science (Cham: Springer), 12033. (2020).

[5] Linares MA Zakaria A. Nizran, P. Skin cancer Prim Care. (2015) 42:645–59. 10.1016/j.pop.2015.07.006 26612377

[6] Anand V Koundal D. Computer-assisted diagnosis of thyroid cancer using medical images: a survey. Lecture Notes in Electrical Engineering (Cham: Springer International Publishing) (2020) p. 543–559.

[7] Narayanan DL Saladi RN Fox JL. Review: ultraviolet radiation and skin cancer: UVR and skin cancer. Int J Dermatol. (2010) 49:978–86. 10.1111/j.1365-4632.2010.04474.x 20883261

[8] Gloster HM Brodland DG. The epidemiology of skin cancer. Dermatol Surg. (1996) 22:217–26. 10.1111/j.1524-4725.1996.tb00312.x 8599733

[9] Wild CP Weiderpass E Stewart BW. World Cancer Report: Cancer Research for Cancer Prevention. IARC Publications. 39432694

[10] Massone C Di Stefani A Soyer HP. Dermoscopy for skin cancer detection. Curr Opin Oncol. (2005) 17:147–53. 10.1097/01.cco.0000152627.36243.26 15725920

[11] Gill KS Sharma A Anand V Gupta R. Assessing the impact of eight EfficientNetB (0- 7) models for leukemia categorization. In: 2023 International Conference on Artificial Intelligence and Knowledge Discovery in Concurrent Engineering (ICECONF). Chennai: IEEE. (2023) p. 1–5.

[12] Barata C Ruela M Francisco M Mendonça T Marques JS. Two systems for the detection of melanomas in dermoscopy images using texture and color features. IEEE Syst J. (2013) 8:965–79. 10.1109/JSYST.2013.2271540

[13] Huang HW Hsu BWY Lee CH Tseng VS. Development of a lightweight deep learning model for cloud applications and remote diagnosis of skin cancers. J Dermatol. (2020) 48:310–6. 33211346 10.1111/1346-8138.15683

[14] Cassidy B Kendrick C Brodzicki A Jaworek-Korjakowska J Yap MH. Analysis of the ISIC image datasets: Usage, benchmarks and recommendations. Med Image Anal. (2022) 75:102305. 10.1016/j.media.2021.102305 34852988

[15] Liang J. Confusion Matrix: Machine Learning. (2022).

[16] Dorj UO Lee KK Choi JY Lee M. The skin cancer classification using deep convolutional neural network. Multimedia Tools Appl. (2018) 77:9909–24. 10.1007/s11042-018-5714-1

[17] Maron RC Weichenthal M Utikal JS Hekler A Berking C Hauschild A. Systematic outperformance of 112 dermatologists in multiclass skin cancer image classification by convolutional neural networks. Eur J Cancer. (2019) 119:57–65. 31419752 10.1016/j.ejca.2019.06.013

[18] Amin J Sharif A Gul N Anjum MA Nisar MW Azam F Bukhari SA. Integrated design of deep features fusion for localization and classification of skin cancer. Pattern Recogn Lett. (2020) 131:63–70. 10.1016/j.patrec.2019.11.04235985337

[19] Hekler A Kather JN Krieghoff-Henning E Utikal JS Meier F Gellrich FF. Effects of label noise on deep learning-based skin cancer classification. Front Med. (2020) 7:1–7. 10.3389/fmed.2020.00177 32435646 PMC7218064

[20] Mahbod A Schaefer G Wang C Dorffner G Ecker R Ellinger I. Transfer learning using a multi-scale and multi-network ensemble for skin lesion classification. Comp Methods Prog Biomed. (2020) 193:1–9. 10.1016/j.cmpb.2020.105475 32268255

[21] Han SS Moon IJ Lim W Suh IS Lee SY Na JI. Keratinocytic skin cancer detection on the face using region-based convolutional neural network. JAMA Dermatol. (2020) 156:29–37. 10.1001/jamadermatol.2019.3807 31799995 PMC6902187

[22] Masni MA Kim DH Kim TS. Multiple skin lesions diagnostics via integrated deep convolutional networks for segmentation and classification. Comp Methods Prog Biomed. (2020) 190:1–12. 10.1016/j.cmpb.2020.105351 32028084

[23] Polat K Koc KO. Detection of skin diseases from dermoscopy image using the combination of convolutional neural network and one-versus-all. J Artif Intellig Syst. (2020) 2:80–97. 10.33969/AIS.2020.21006

[24] Duggani K Nath MK. A technical review report on deep learning approach for skin cancer detection and segmentation. Data Analyt Manage. (2021) 54:87–99. 10.1007/978-981-15-8335-3_936774890

[25] Khan MA Zhang YD Sharif M Akram T. Pixels to classes: intelligent learning framework for multiclass skin lesion localization and classification. Comp Elect Eng. (2021) 90:1. 10.1016/j.compeleceng.2020.106956

[26] Shetty B Fernandes R Rodrigues AP Chengoden R Bhattacharya S Lakshmanna K. “Skin lesion classification of dermoscopic images using machine learning and convolutional neural network,” Sci. Rep, vol 12, no. (2022) 1:18134. 10.1038/s41598-022-22644-9 36307467 PMC9616944

[27] Anand V Gupta S Altameem A Nayak SR Poonia RC Saudagar AKJ. An enhanced transfer learning based classification for diagnosis of skin cancer. Diagnostics (Basel). (2022) 12:7. 10.3390/diagnostics12071628 35885533 PMC9316548

[28] Anand V Gupta S Koundal D Nayak SR Nayak J Vimal S. Multiclass skin disease classification using transfer learning model. Int J Artif Intell Tools. (2022) 31:2. 10.1142/S0218213022500294

[29] Aldhyani THH Verma A Al-Adhaileh MH Koundal D. Multiclass skin lesion classification using a lightweight dynamic kernel deep-learning-based convolutional neural network. Diagnostics (Basel). (2022) 12:2048. 10.3390/diagnostics12092048 36140447 PMC9497471

[30] Nigar N Umar M Shahzad MK Islam S. Abalo D. A deep learning approach based on explainable artificial intelligence for skin lesion classification. IEEE Access. (2022) 10:113715–25. 10.1109/ACCESS.2022.3217217

[31] Dhiman G Juneja S Viriyasitavari W Mohafez H Hadizadeh M Islam M. A novel machine-learning-based hybrid CNN model for tumor identification in medical image processing. Sustainability. (2022) 14:1447. 10.3390/su14031447

[32] Aggarwal S Gupta S Gupta D Gulzar Y Juneja S Alan A. An artificial intelligence-based stacked ensemble approach for prediction of protein subcellular localization in confocal microscopy images. Sustainability. (2023) 15:1695. 10.3390/su15021695

